# Complete Genome Sequence of *Komagataeibacter hansenii* LMG 23726^T^

**DOI:** 10.1128/genomeA.00168-17

**Published:** 2017-04-13

**Authors:** Sarah Pfeffer, Richard Santos, Marcus Ebels, Darius Bordbar, R. Malcolm Brown

**Affiliations:** Molecular Biosciences, College of Natural Sciences, University of Texas at Austin, Austin, Texas, USA

## Abstract

This study reports the release of the complete nucleotide sequence of* Komagataeibacter hansenii* LMG 23726^T^. This organism is a cellulose producer, and its genome may provide more information to aid in the understanding of the genes necessary for cellulose biosynthesis.

## GENOME ANNOUNCEMENT

*Komagataeibacter hansenii* LMG 23726^T^, previously known as *Gluconacetobacter hansenii*, is a Gram-negative, nitrogen-fixing, cellulose-producing, rod-shaped cell that was isolated from Kombucha tea (1, 2). This strain is closely related to other *Komagataeibacter* strains that are particularly efficient producers of pure, highly crystalline cellulose known as bacterial cellulose (BC) (1 – 5). BC is synthesized through pores along the long axis of the cell which associate in a hierarchical cell-directed self-assembly process to form ribbons and ultimately a membrane located at the air–liquid interface (6 – 8). Because of its ultrafine reticulated structure, high crystallinity, great mechanical strength, high water-holding capacity, moldability during formation, and biocompatibility, BC is well suited for medical, industrial, and commercial applications (9 – 12). The purpose of this report is to contribute to the data available to provide further insight into the molecular mechanisms of bacterial cellulose biosynthesis and add to the study of *Gluconacetobacter* spp.

Here, we present the genome of *Komagataeibacter hansenii* strain LMG 23726^T^ (BCCM). The DNA was extracted and subjected to sequencing using an Illumina HiSeq 2000 PE100 system (University of Texas at Austin, ICMB Core Facility). The reads were downloaded into Geneious version 8.1.2 and assembled into contigs using Velvet version 1/2/02 (13), where it was revealed that the genome is approximately 3.6 Mb in size with a G+C content of 59.3% (14). A total of 3,733 open reading frames were predicted using Glimmer (15). Annotation data on contigs containing cellulose synthase genes were determined.

Preliminary phylogenetic analysis using 16S rRNA genes determined that this new strain is closely related to *K. hansenii* ATCC 23769. A homology comparison to the *acsABCD* operon of *K. hansenii* ATCC 23769 (GenBank accession no. AB091060) was performed and resulted in a 99.8% identity to *acsAB*, 99.5% identity to *acsC*, and 100% identity to *acsD*. Further investigations into the genome indicated that *K. hansenii* LMG 23726^T^ contains a total of three separate coding regions for cellulose biosynthesis: *acsABCD*, *acsAII*, and *acsABC*. These three operons are also found in *K. hansenii* ATCC 23769. A homology comparison of the shared cellulose-synthesizing regions revealed a sequence identity of 74.5% identity to *acsAII* and 99.6% identity to *acsABC*. The *acsABCD* operon is flanked by genes coding for proteins which have been determined to be essential for proper cellulose biosynthesis to occur: *cmcAx, ccpAx*, and* bglAx* (16 – 19). These genes shared, respectively, 99.7%, 98.6%, and 99.3% sequence identities to *K. hansenii* ATCC 23769.

Further investigations into the genome of *K. hansenii* LMG 23726^T^ may provide more insight into the mechanisms necessary for cellulose biosynthesis.

### Accession number(s).

This whole-genome shotgun project has been deposited in DDBJ/ENA/GenBank under the accession number MJMD00000000. The version described in this paper is the first version, MJMD01000000.
